# Intermittent fasting exacerbates microplastic-induced gut inflammation via *Rikenella*-mediated regulation of purine metabolism and Th17/Treg balance

**DOI:** 10.1038/s41522-026-01016-5

**Published:** 2026-06-15

**Authors:** Rui Fu, Yixian Cheng, Junjie Chen, Weiwei Sheng, Jinghua Gu, Jiahao Wang, Zheng Zhang, Huake Cao, Jiawei Zhang, Bo Chen, Guodong Cao

**Affiliations:** Department of General Surgery, First Affiliated Hospital of Anhui Medical University, Hefei City, PR China

**Keywords:** Environmental sciences, Immunology, Microbiology

## Abstract

The pervasive accumulation of microplastics (MPs) in ecosystems and human bodies has prompted growing concern regarding their biological effects. However, whether intermittent fasting (IF) modulates MP-induced gut injury remains unclear. We established a murine model of chronic oral exposure to 1 μm polystyrene MPs combined with a daily 8-h feeding window. Multi-omics analyses, including 16S rRNA sequencing, untargeted metabolomics, and immune profiling, were performed. In a murine model of chronic oral exposure to 1 μm polystyrene microplastics, IF aggravated colonic inflammation and oxidative stress. 16S rRNA sequencing showed that IF further altered the microplastic-associated gut microbiota, notably reducing *Rikenella* abundance. Untargeted metabolomics revealed disrupted purine metabolism, including decreased hypoxanthine levels, which positively correlated with *Rikenella*. Combined MP and IF exposure was associated with an increased Th17/Treg ratio. Supplementation with *Rikenella* or hypoxanthine partially attenuated inflammatory changes. These findings unveil a previously unappreciated risk of IF under conditions of environmental microplastic exposure, and highlight the necessity of integrating environmental toxicants into evaluations of dietary interventions.

**Figure:**
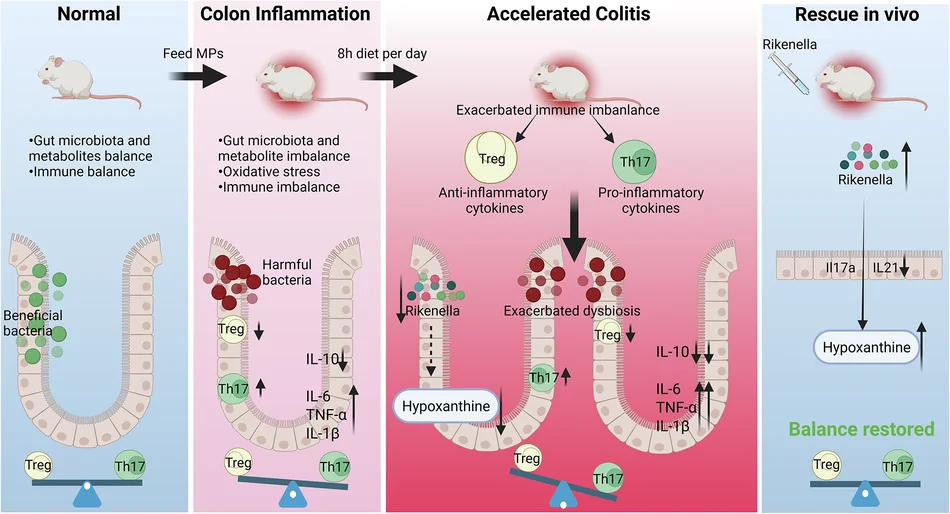

## Background

Microplastics (MPs), defined as synthetic polymer particles smaller than 5 mm, are now ubiquitously present across marine, terrestrial, and atmospheric environments. Human exposure to microplastics likely occurs mainly through ingestion of contaminated food and water. While microplastics have been reported in human biological samples, interpretation of these findings is limited by methodological concerns. Experimental animal studies, however, have demonstrated uptake after oral exposure and subsequent tissue distribution^1,2^. These findings demonstrate that MPs can traverse biological barriers and accumulate in various organs. Due to their physicochemical stability and resistance to degradation, MPs persist in biological systems and elicit a range of harmful responses, including oxidative stress, immune dysregulation, and chronic inflammation factors implicated in the pathogenesis of tissue injury, fibrosis, and tumorigenesis^3–5^. Despite growing concern, the in vivo toxicodynamics of MPs and their long-term effects on host physiology remain poorly understood.

The gastrointestinal tract is the primary portal of entry for MPs and is thus a key site for their biological interaction. Experimental models have shown that chronic MP exposure disrupts gut microbial composition, impairs intestinal barrier function, and induces mucosal inflammation^6^. MPs vary in their size, shape, and polymer type, yet consistently perturb microbial homeostasis, reduce beneficial commensals, and increase pathobionts^7^. Histologically, MP exposure is associated with epithelial damage and increased expression of pro-inflammatory cytokines. Although the mucus layer provides partial protection by trapping ingested particles, smaller MPs can penetrate this barrier and translocate into deeper tissues, raising the risk of systemic dissemination^8,9^. However, existing research largely focuses on acute or high-dose exposures^10^, and the chronic impacts of low-dose MP ingestion, especially under physiologically relevant conditions remain insufficiently characterized.

Intermittent fasting (IF), particularly in the form of time-restricted feeding, is increasingly recognized for its systemic benefits, including improved metabolic efficiency, reduced oxidative stress, and enhanced immune homeostasis^11,12^. IF also exerts significant effects on the intestinal microenvironment by reshaping gut microbiota and modulating metabolite profiles^13,14^. Previous studies have reported that IF increases the abundance of mucin-degrading bacteria such as *Akkermansia muciniphila*, enhances gut barrier integrity, and alters mucosal immune responses^15^. However, the influence of IF on host responses under conditions of environmental toxicant exposure is not well defined, and the interaction between IF and chronic MP exposure has not been systematically investigated.

This study establishes a murine model of chronic MP exposure combined with an 8-h daily feeding window to examine the impact of IF on MP-induced intestinal injury. Multi-omics approaches including 16S rRNA gene sequencing, untargeted metabolomics, and immunophenotyping were employed to characterize changes in gut microbial composition, metabolic pathways, and immune regulation. The findings demonstrate that IF amplifies MP-induced gut inflammation by promoting microbial dysbiosis, disrupting purine metabolism via contraction of *Rikenella*, and skewing the Th17/Treg balance toward a pro-inflammatory state. IF also enhances MP bioaccumulation and oxidative stress, contributing to further compromise of intestinal barrier integrity. These results reveal a previously unrecognized interaction between dietary pattern and environmental exposure, and underscore the need to consider environmental context when evaluating the health effects of nutritional interventions.

## Results

### Characterization of PS MPs and their uptake by intestinal epithelial cells

**Fig. 1:**
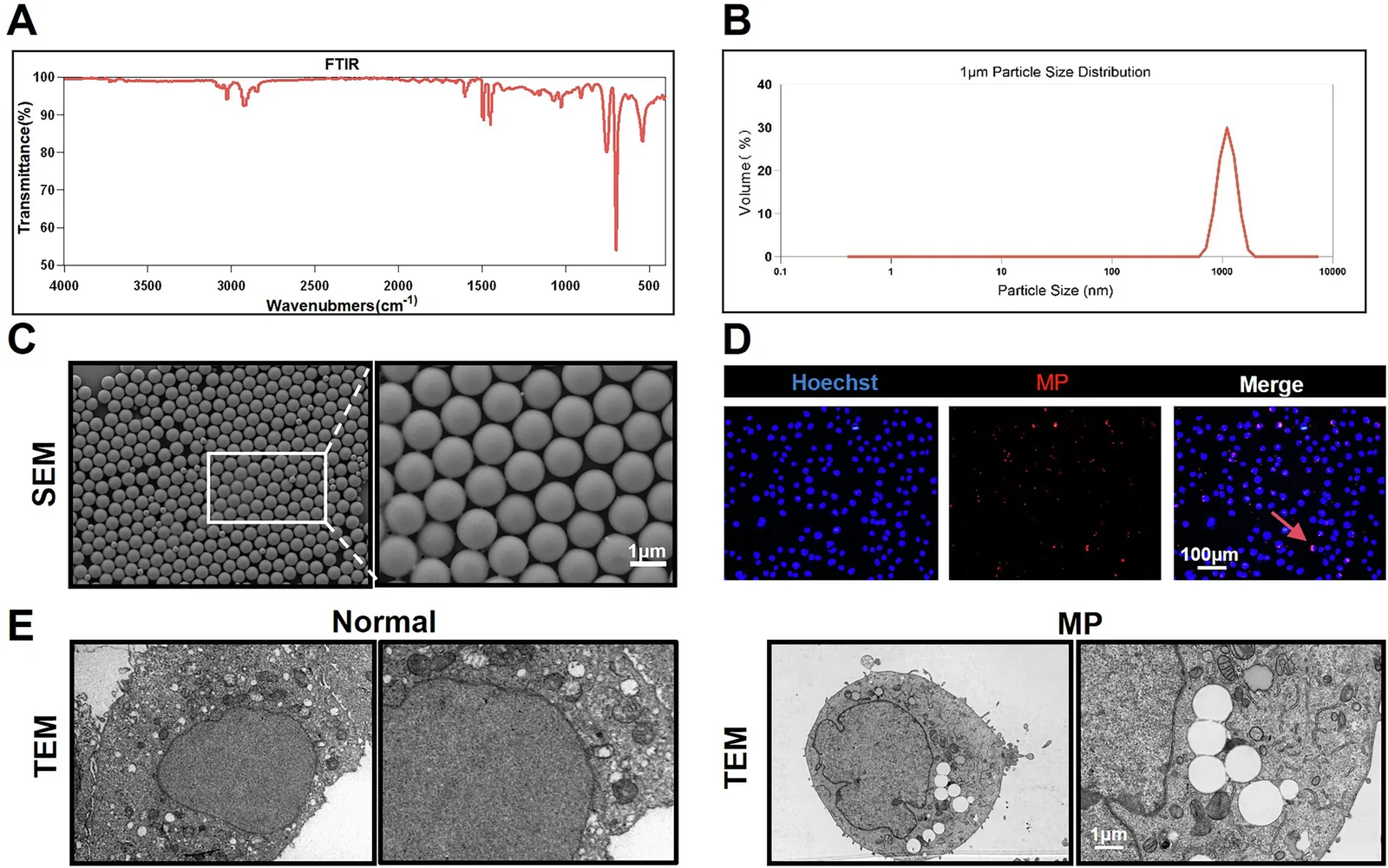
Characterization of PS MPs and their uptake by intestinal epithelial cells. **A** Fourier-transform infrared spectroscopy (FTIR) analysis confirms the chemical composition of the PS MPs. **B** Particle size distribution of PS MPs measured by laser diffraction indicates a predominant diameter of 1 μm. **C** Scanning electron microscopy reveals that the PS MPs possess a uniform spherical morphology with high monodispersity. **D** Fluorescence microscopy demonstrates the internalization of PS MPs by colonic epithelial cells. **E** Transmission electron microscopy reveals structural damage to mitochondria following MP internalization: mitochondrial swelling, outer membrane irregularity, and inner membrane disruption were observed 24 h post-exposure, while the plasma membrane remained largely intact.

To confirm the chemical identity of the microplastic samples used in this study, Fourier-transform infrared (FTIR) spectroscopy was performed. The resulting spectra exhibited characteristic absorption peaks consistent with PS, confirming the polymeric composition of the particles (Fig. 1A). Particle size distribution analysis using laser diffraction revealed a narrow distribution centered around 1 μm, indicating a high degree of size uniformity (Fig. 1B). Scanning electron microscopy (SEM) further confirmed the morphological features of the PS MPs, demonstrating spherical structures with an average diameter of approximately 1 μm and high monodispersity (Fig. 1C). These results collectively validated the physicochemical uniformity of the PS MPs, confirming their suitability for subsequent biological experiments.

To assess whether PS MPs could be internalized by intestinal epithelial cells, we co-cultured 1 μm fluorescently labeled PS MPs with colonic epithelial cells in vitro. After 24 h of incubation, fluorescence microscopy revealed robust internalization of the particles by the epithelial cells (Fig. 1D). Transmission electron microscopy (TEM) provided ultrastructural confirmation of intracellular localization of the PS MPs, as well as evidence of subcellular damage. Specifically, mitochondria exhibited marked swelling, disrupted outer membranes, and deformation or rupture of cristae, indicative of organelle-level stress. In contrast, the plasma membrane remained largely intact, suggesting selective intracellular vulnerability following particle uptake (Fig. 1E). These findings establish that 1 μm PS MPs are not only well-characterized and monodisperse but are also readily internalized by colonic epithelial cells, where they induce distinct morphological changes at the organelle level.

### IF exacerbates MPs induced intestinal inflammation

**Fig. 2:**
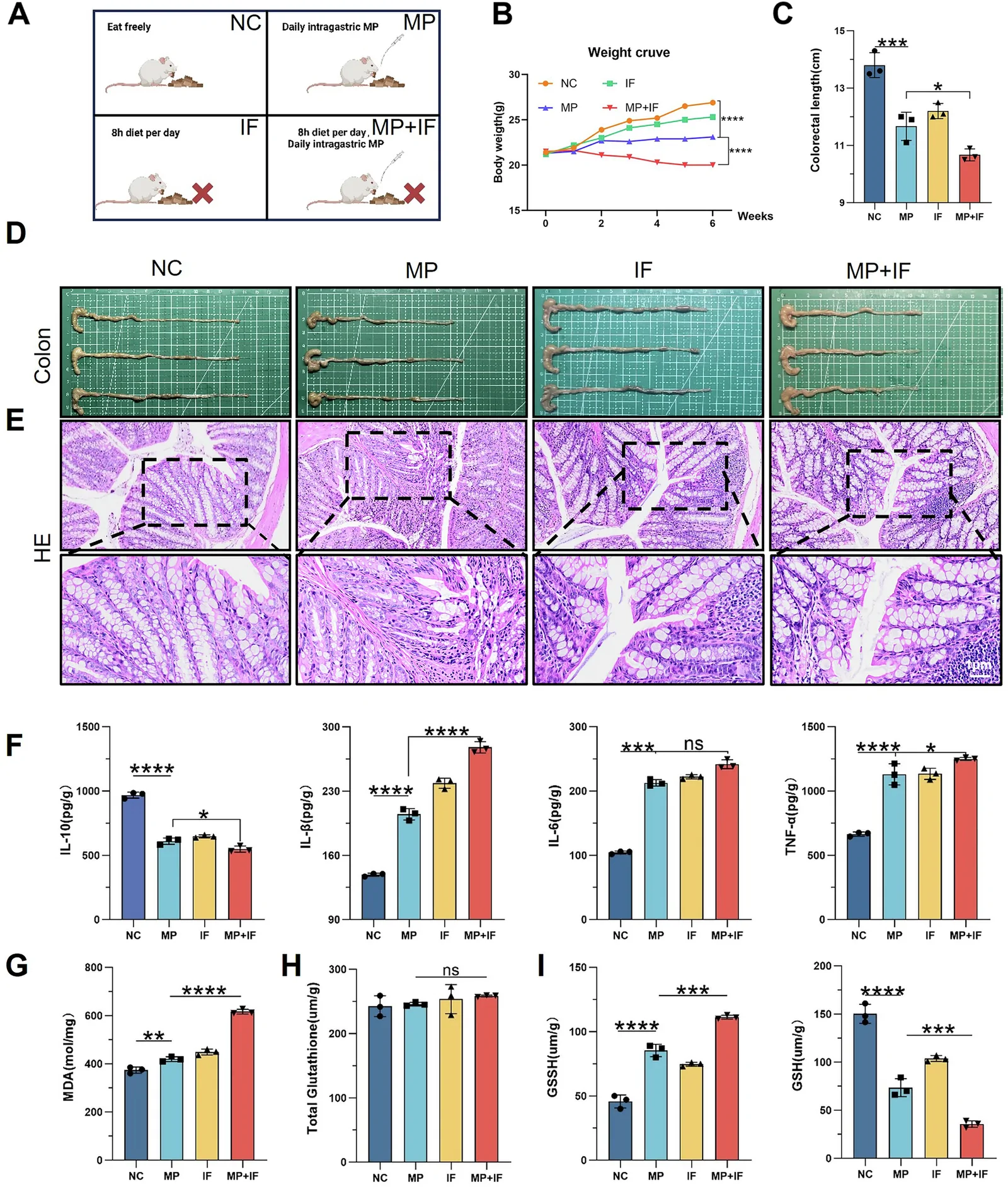
Intermittent fasting exacerbates microplastic-induced intestinal inflammation. After 1 week of acclimation, mice were randomly assigned to four groups: NC, MP, IF, and MP + IF (*n* = 3 mice per group). Mice in the MP and MP + IF groups were administered 1 μm polystyrene microplastics (PS-MPs) by oral gavage at a dose of 10 mg/kg body weight/day for 6 consecutive weeks, whereas mice in the NC and IF groups received an equal volume of PBS. Mice in the IF and MP + IF groups were subjected to time-restricted feeding with an 8 h feeding window per day, with free access to water throughout the experiment. Body weight was recorded every 2 days during the treatment period. At the end of the experiment, mice were fasted for 8 h, euthanized, and colon tissues were collected for further analysis. **A** Schematic diagram of the animal experimental protocol outlining MP exposure and IF regimens over 6 weeks. **B** Body weight changes of mice during the experimental period under different treatment conditions. **C**, **D** Representative images and quantitative analysis of colon length following treatment. **E** H&E staining reveals increased inflammatory cell infiltration in the colonic mucosa of IF-treated mice exposed to MPs. **F** ELISA quantification of colonic cytokines shows increased pro-inflammatory markers (TNF-α, IL-1β) and decreased anti-inflammatory cytokine (IL-10) levels in the MP + IF group. For the MP and MP+IF groups, there was no significant change in IL-6. **G** Levels of malondialdehyde (MDA), a marker of lipid peroxidation, are significantly elevated in colonic tissues of MP + IF mice. **H**, **I** Oxidative stress indicators—glutathione disulfide (GSSG) and reduced glutathione (GSH) demonstrate redox imbalance in the MP + IF group. Three independent biological replicates were analyzed in each group. Data are presented as mean ± s.e.m. Statistical significance was determined using one-way ANOVA with multiple comparisons: *p < 0.05, **p < 0.01, ***p < 0.001, ****p < 0.0001.

Chronic exposure to MPs has been shown to induce intestinal inflammation and tissue injury, while dietary interventions such as IF are known to modulate inflammatory responses^16,17^. To assess the interaction between IF and MP-induced intestinal injury, we implemented a time-restricted feeding regimen (8-h feeding window per day) in mice chronically exposed to 1 μm polystyrene MPs. Over the 6-week exposure period, mice subjected to both MP and IF exhibited significantly lower body weight compared to those exposed to MPs alone, indicating a physiological impact of IF under MP exposure conditions (Fig. 2B). Gross anatomical examination revealed a marked reduction in colon length in the MP group relative to controls, consistent with inflammation-associated tissue contraction. Notably, colon shortening was more pronounced in the group exposed to both MP and IF, with a further significant reduction compared to MP exposure alone (*P* < 0.05; Fig. 2C, D).

Histological analysis of H&E stained colon sections revealed extensive infiltration of inflammatory cells in the combined MP and IF exposure group, exceeding that observed in either the MP or IF groups alone (Fig. 2E). This exacerbated histopathology was accompanied by significant alterations in cytokine profiles. Enzyme-linked immunosorbent assays showed elevated levels of pro-inflammatory cytokines TNF-α(*P* < 0.05), IL-1β(*P* < 0.0001) in the group subjected to both MP and IF, while the anti-inflammatory cytokine IL-10 was markedly decreased (*P* < 0.05; Fig. 2F). These data indicate that IF intensifies the inflammatory response initiated by MP exposure, compared to the group subjected to MP only, rather than mitigating it. To investigate whether oxidative stress contributed to the observed inflammation, we quantified redox markers in colon tissue. MP exposure alone increased levels of malondialdehyde (MDA, *P* < 0.0001) and oxidized glutathione (GSSG, *P* < 0.001), while significantly reducing reduced glutathione (GSH, *P* < 0.001), indicating elevated oxidative stress (Fig. 2G–I). Importantly, these alterations were further amplified by combined exposure to MP and IF, which led to even higher MDA and GSSG levels and lower GSH concentrations, consistent with aggravated oxidative damage. Collectively, these results demonstrate that IF, when co-administered with chronic MP exposure, does not confer protection but instead exacerbates intestinal inflammation and oxidative stress. This suggests a detrimental interaction between dietary restriction and environmental pollutant exposure, with significant implications for gut health.

### Single-cell transcriptomics suggests Th17-associated skewing of intestinal T cells following MPs exposure

**Fig. 3:**
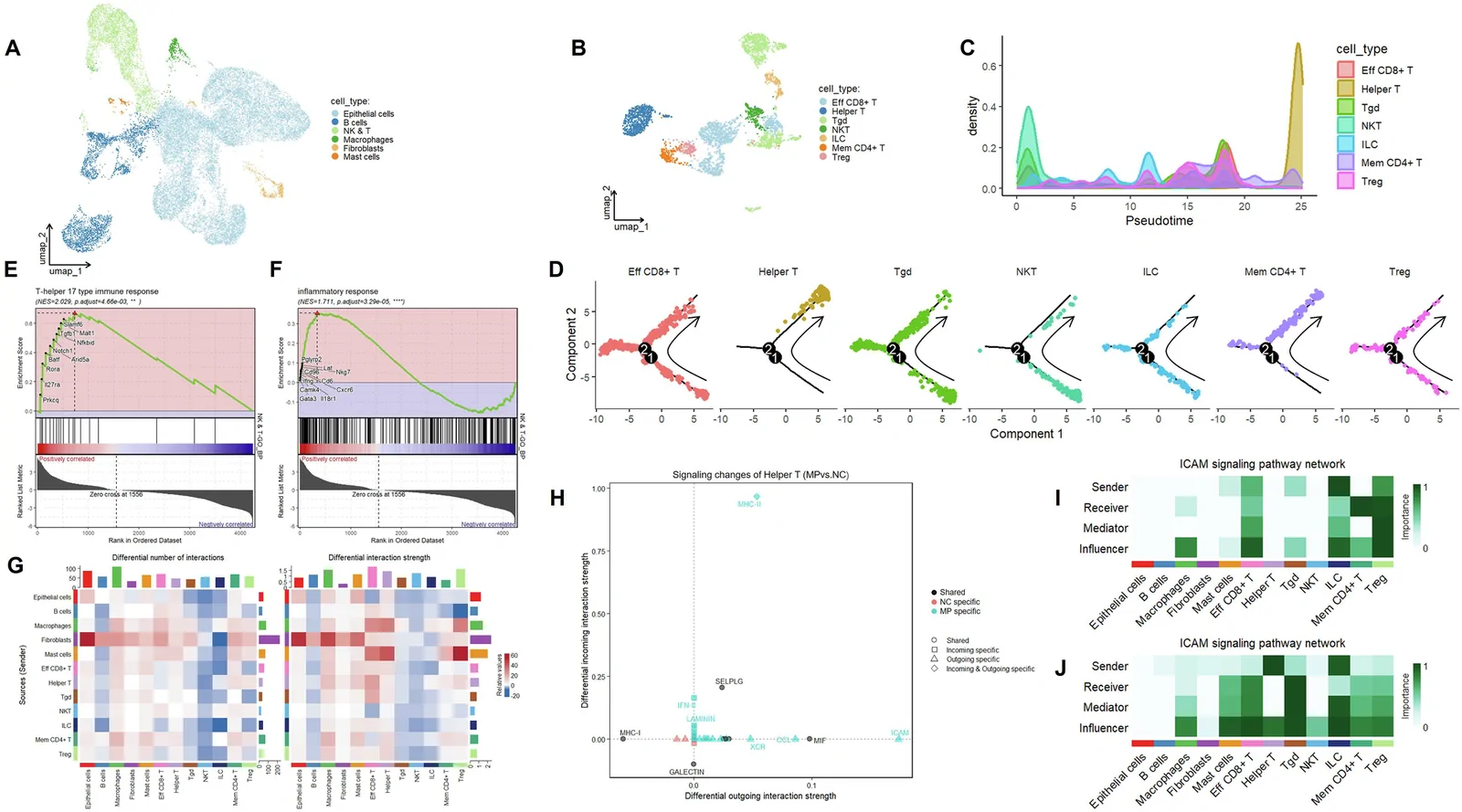
The effect of microplastics on mouse intestinal T cells. **A** Single-cell atlas of mouse intestinal cells. **B** Single-cell atlas of mouse intestinal T cells. **C**, **D** Trajectory of mouse intestinal T cells. **C** The dynamic changes of T cell subtype abundance along the time axis. **D** The distribution of different T cell subtypes along the cell trajectory. **E**, **F** GO_BP pathway GSEA enrichment analysis of characteristic genes in mouse intestinal T cell populations. **G** Differential interaction quantities or intensity under different conditions. The colored bar charts at the top of the heatmap represent the sum of absolute values for each column (incoming signals), while the colored bar chart on the right reflects the sum of absolute values for each row (outgoing signals). The height of the bars indicates the degree of change in interaction quantity or intensity between the two conditions. In the heatmap body, red indicates enhanced signal strength in the MP group compared to the UT group, while blue indicates reduced signal strength. **H** Differential cell-cell communication pathways between Helper T Cells in the NC and MP Groups. Cell Chat Identifies Key Signal Senders, Receivers, Mediators, and Regulators in intercellular communication networks by calculating multiple centralitymeasures for each cell population in the NC (**I**) and MP (**J**) groups.

To investigate the immune landscape of the intestine under MPs exposure, we analyzed publicly available single-cell RNA sequencing data from intestinal tissues of MP-treated mice. Integration and clustering of the transcriptomic profiles generated a comprehensive intestinal cell atlas, revealing notable shifts in immune cell composition. Specifically, MP treatment was associated with an increased proportion of T cells and natural killer (NK) cells (Fig. 3A; Fig. S1A, B). Cell trajectory analysis indicated that helper T (Th) cells were enriched at the terminal state of the pseudotime trajectory, consistent with commitment toward effector phenotypes (Fig. 3B–D; Fig. S1C). Gene set variation analysis revealed significant enrichment of general inflammatory pathways (NES = 2.029, adjusted *P* < 0.001) and the Th17 differentiation program (NES = 1.711, adjusted *P* < 0.001), suggesting a shift in Th cell states toward Th17-associated transcriptional profiles (Fig. 3E, F), rather than providing direct evidence of dynamic differentiation.

In parallel, intercellular communication analysis showed altered signaling networks within the intestinal microenvironment following MPs exposure. Enhanced crosstalk was observed among multiple immune lineages, with Th cells exhibiting pronounced upregulation in ICAM (intercellular adhesion molecule) signaling (Fig. 3G–J; Fig. S2A–E). This pathway plays a key role in immune cell adhesion, trafficking, and activation, and may contribute to the observed Th17-associated inflammatory responses^18–21^.

### IF exacerbates MPs-induced intestinal inflammation via Th17/Treg imbalance

**Fig. 4:**
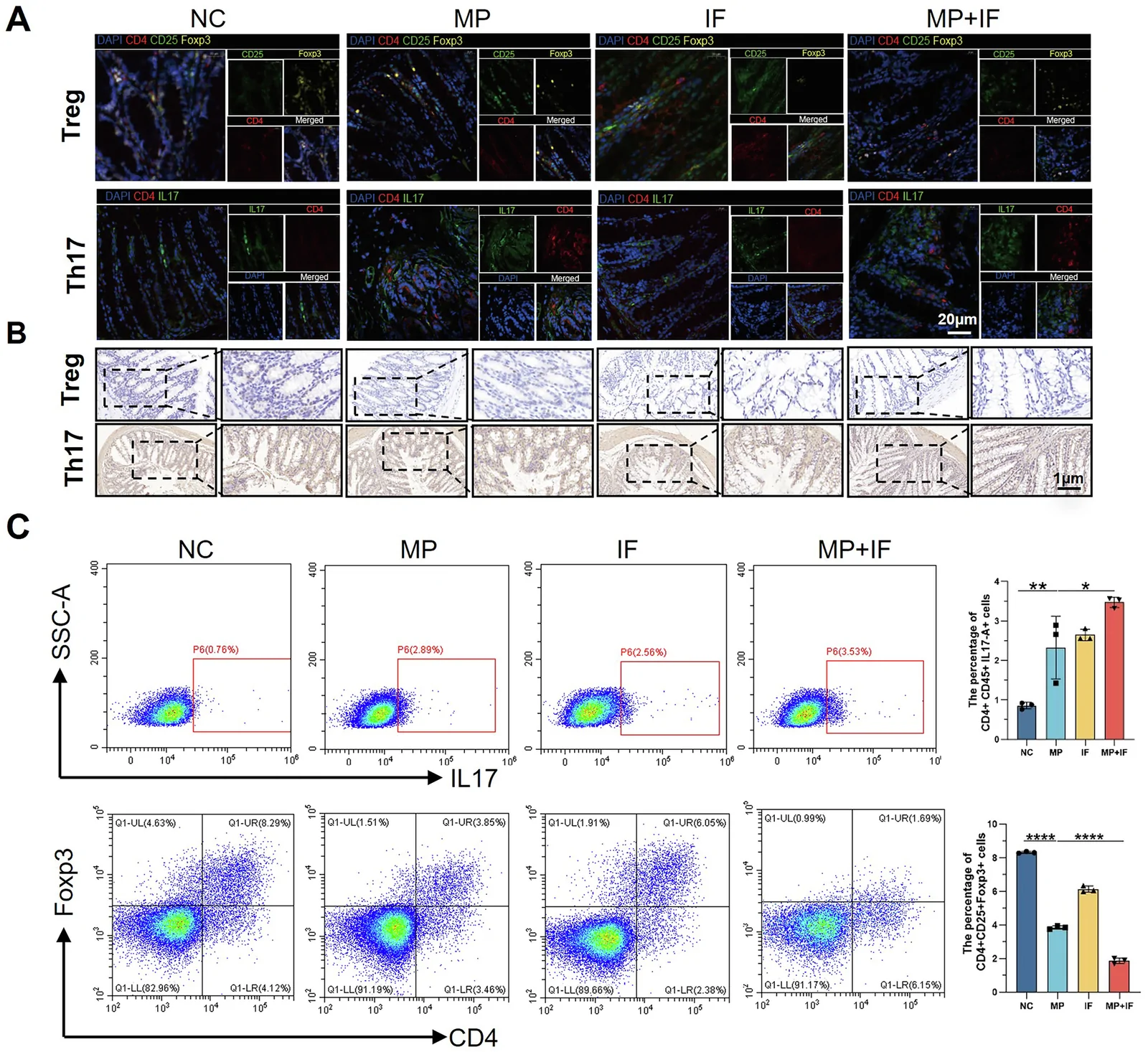
Intermittent fasting causes an imbalance in the Th17/Treg ratio, promoting microplastic-induced intestinal inflammation. After 1 week of acclimation, mice were randomly assigned to four groups: NC, MP, IF, and MP + IF (*n* = 3 mice per group). Mice in the MP and MP + IF groups were administered 1 μm MPs by oral gavage at a dose of 10 mg/kg body weight/day for 6 consecutive weeks, whereas mice in the NC and IF groups received an equal volume of PBS. Mice in the IF and MP + IF groups were subjected to time-restricted feeding with an 8 h feeding window per day, with free access to water throughout the experiment. At the end of the experiment, mice were fasted for 8 h, euthanized, and intestinal tissues and spleens were collected for analysis. **A** Multiplex immunofluorescence of Th17 and Treg cells, the MP + IF group displayed higher Th17 cell infiltration but lower Treg cell levels compared to the MP group. **B** Immunohistochemical measurement of Th17 and Treg cells, the MP + IF group showed decreased Foxp3 but increased IL-17 expression compared with MP group. **C** Determination of the content of Th17 and Treg cells in the spleen by flow cytometry, the MP + IF group displayed higher Th17 cell infiltration but lower Treg cell levels compared to the MP group. Three biologically independent mice were analyzed in each group. Data are presented as mean ± s.e.m. Statistical significance was determined using one-way ANOVA with multiple comparisons: *p < 0.05, **p < 0.01, ***p < 0.001, ****p < 0.0001.

Th17 and regulatory T (Treg) cells are functionally antagonistic subsets that play a pivotal role in maintaining intestinal immune homeostasis. A balanced Th17/Treg ratio is essential for mucosal tolerance and inflammation control, while disruption of this equilibrium is associated with chronic inflammatory conditions such as inflammatory bowel disease^22^. Previous studies suggest that MPs can directly interact with intestinal macrophages and immune cells, promoting Th17 polarization and contributing to immune dysregulation^23^. To explore whether IF exacerbates MP-induced inflammation through alteration of the Th17/Treg axis, we performed multiplex immunofluorescence staining of intestinal tissues. Representative images showed a trend toward reduced CD4^+^CD25^+^Foxp3^+^ Treg staining and increased CD4^+^IL-17A^+^ Th17 staining in MP-treated mice compared with controls. This trend appeared more evident in the MP+IF group (Fig. 4A). Histological analysis revealed aggravated inflammatory injury, which was consistent with the immunofluorescence observations (Fig. 4B).

To further assess systemic immune responses, we performed flow cytometry on splenic T cells from each group. Quantitative analysis revealed a stepwise increase in the proportion of CD4^+^IL-17A^+^ Th17 cells across experimental conditions, with the lowest levels observed in the control group, followed by the MP-treated group, and the highest levels detected in the group subjected to both MP exposure and IF. Conversely, CD4⁺CD25⁺Foxp3⁺ Treg cell frequencies exhibited a progressive decline across these groups. Specifically, Th17 levels in both MP exposure and IF group were 0.6% higher than in the MP group, which itself was 2% higher than in controls. Conversely, CD4⁺CD25⁺Foxp3⁺ Treg cells showed a stepwise decline, with both MP exposure and IF group exhibiting a 2% reduction compared to the MP group, and the MP group 4% lower than controls (Fig. 4C). These changes resulted in a significantly elevated Th17/Treg ratio in both MP exposure and IF group (Fig. S3A), highlighting a shift toward a pro-inflammatory immune profile. Together, these results demonstrate that IF amplifies MP-induced immune dysregulation by further skewing the Th17/Treg balance toward Th17 dominance. This imbalance likely contributes to the exacerbation of intestinal inflammation and underscores a critical interplay between environmental toxicants and dietary interventions in modulating mucosal immunity.

### IF alters gut microbiota composition and exacerbates MP-induced inflammation via *Rikenella* depletion

**Fig. 5:**
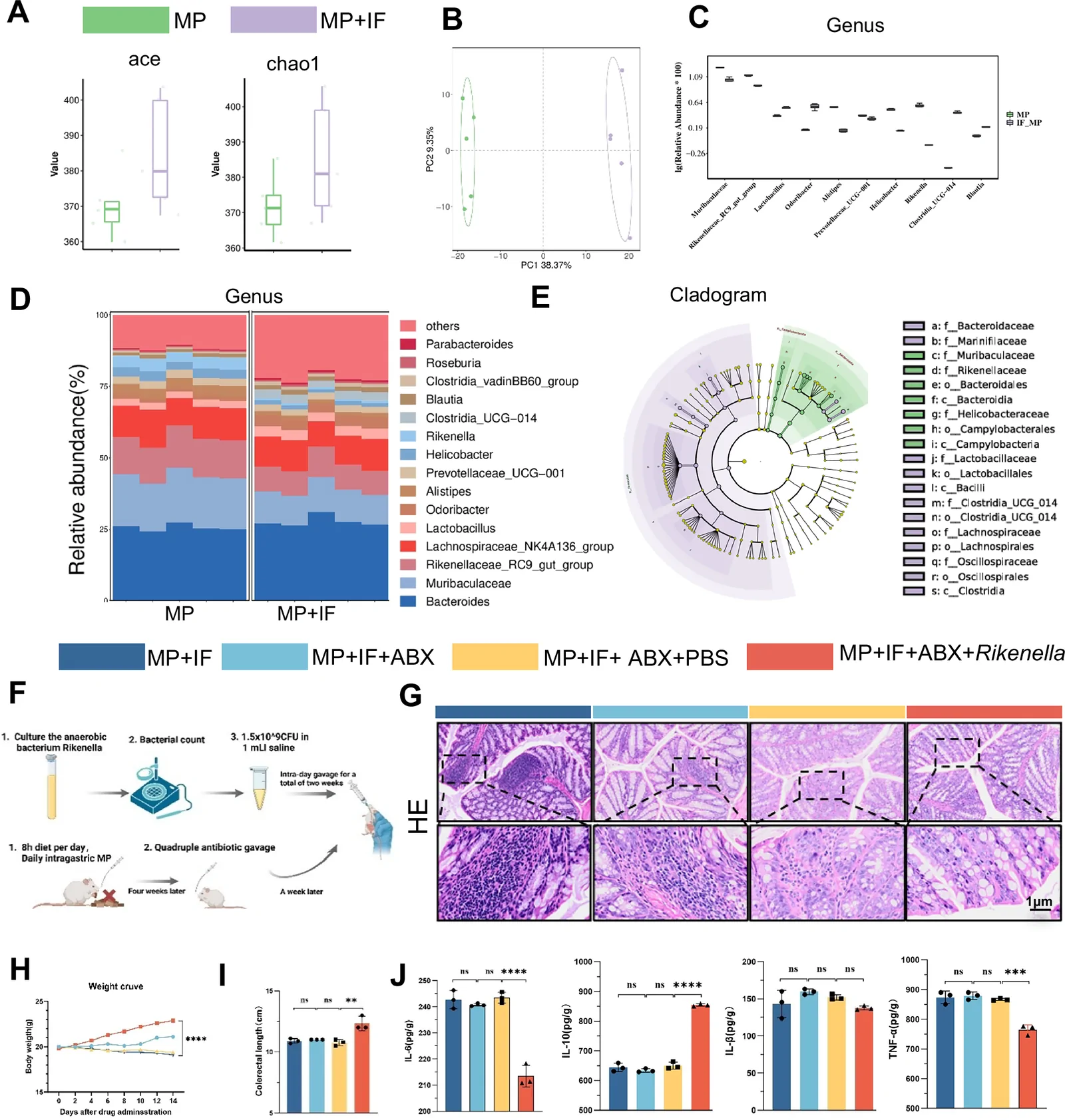
16S rRNA analysis reveals that intermittent fasting exacerbates microplastic-induced intestinal inflammation via *Rikenella depletion.* For microbiota analysis, mice in the MP and MP + IF groups were exposed to 1 μm polystyrene MPs by oral gavage at a dose of 10 mg/kg body weight/day for 6 consecutive weeks. Mice in the MP + IF group were additionally subjected to time-restricted feeding with an 8 h feeding window per day, with free access to water. Intestinal contents were collected for 16S rRNA gene sequencing (*n* = 5 mice per group). Then, after establishment of the MP + IF mouse model, mice were treated with a broad-spectrum antibiotic cocktail by gavage for 7 consecutive days to deplete the gut microbiota. One week later, mice were orally administered *Rikenella* (100 μL/mouse, 1.5 × 10^9^ CFU/mL) or an equal volume of PBS 3 times per week for 2 weeks. **A** Analysis of α diversity between the MP group and MP + IF group, ACE and Chao1 indexes, showed no significant differences. **B** β diversity between the MP group and the MP + IF group, There is a significant difference between the two groups. **C** Top 10 box plots of differential microflora at the genus level. **D** Community structure at the genus level was used to analyze the relative abundance of different bacteria in the two groups of samples. **E** LEFSe analysis. **F** Diagram of mouse modeling (*n* = 3 per group). **G** H&E staining showed that inflammatory cell infiltration was reduced in the colonic mucosa of mice treated with *Rikenella*. **H** Body weight changes of ABX mice in each group after feeding *Rikenella* bacteria. **I** Representative images and quantitative analysis of colon length following treatment. **J** ELISA quantification of colonic cytokines showed that the levels of pro-inflammatory markers (TNF-α, IL-6) were reduced and the level of anti-inflammatory cytokine (IL-10) was increased in the *Rikenella*-treated group, IL-1β showed no significant change. Three biologically independent mice were analyzed in each group. Data are presented as mean ± s.e.m. Statistical significance was determined using one-way ANOVA with multiple comparisons: *p < 0.05, **p < 0.01, ***p < 0.001, ****p < 0.0001.

Accumulating evidence underscores the central role of the gut microbiota in regulating mucosal immunity and shaping host inflammatory responses^24,25^. Inflammatory processes in the gut are frequently accompanied by compositional shifts in the microbial community, which may potentiate disease progression^26^. To explore whether IF modulates the gut microbiota in the context of MPs exposure, we performed 16S rRNA gene sequencing on intestinal contents collected from mice exposed to MPs alone and from those subjected to combined MPs and IF (*n* = 5 per group). Alpha-diversity, assessed via ACE and Chao1 indices, showed no significant differences between the two groups (Fig. 5A). However, principal coordinate analysis (PCoA) of beta-diversity revealed a distinct separation in microbial composition, indicating that IF altered the MP-induced microbial community structure (Fig. 5B). Taxonomic profiling at the genus level showed marked depletion of beneficial taxa including *Muribaculaceae*, *Lachnospiraceae*, and *Rikenella* in the group exposed to both MPs and IF (Fig. 5D). At the genus level, differential abundance analysis identified a significant reduction in *Rikenella* in the combined exposure group, as confirmed by relative abundance boxplots (Fig. 5C) and LEfSe analysis, which revealed 32 taxa with significantly altered abundance, including *Rikenella* (Fig. 5E).

Given prior evidence suggesting a regulatory role of *Rikenella* in intestinal immune homeostasis and metabolic function, we conducted follow-up animal experiments to assess its mechanistic involvement^27^. Mice subjected to broad-spectrum antibiotic treatment (ABX group) displayed minimal weight changes over 6 weeks, consistent with microbiota depletion affecting both pathogenic and symbiotic species. Notably, restoration of *Rikenella* through oral gavage led to a significant increase in body weight compared to the ABX + PBS group (Fig. 5G). Upon necropsy, the *Rikenella*-supplemented group exhibited significantly longer colon lengths (*P* < 0.01), suggesting amelioration of MP -induced tissue damage (Fig. 5H). Histological examination revealed reduced inflammatory cell infiltration in the *Rikenella*-treated group relative to controls (Fig. 5I). Cytokine profiling via ELISA further demonstrated a pronounced anti-inflammatory shift: levels of TNF-α (*P* < 0.001), and IL-6 (*P* < 0.0001) were significantly decreased, while IL-10 was markedly elevated (*P* < 0.0001) (Fig. 5J). Collectively, these results indicate that IF exacerbates MP-induced intestinal inflammation in part by depleting protective microbial taxa such as *Rikenella*, which appears to mitigate inflammation through cytokine modulation and preservation of gut barrier integrity.

### Transcriptomic profiling indicates that *Rikenella* attenuates inflammation via modulation of the Th17/Treg axis

**Fig. 6:**
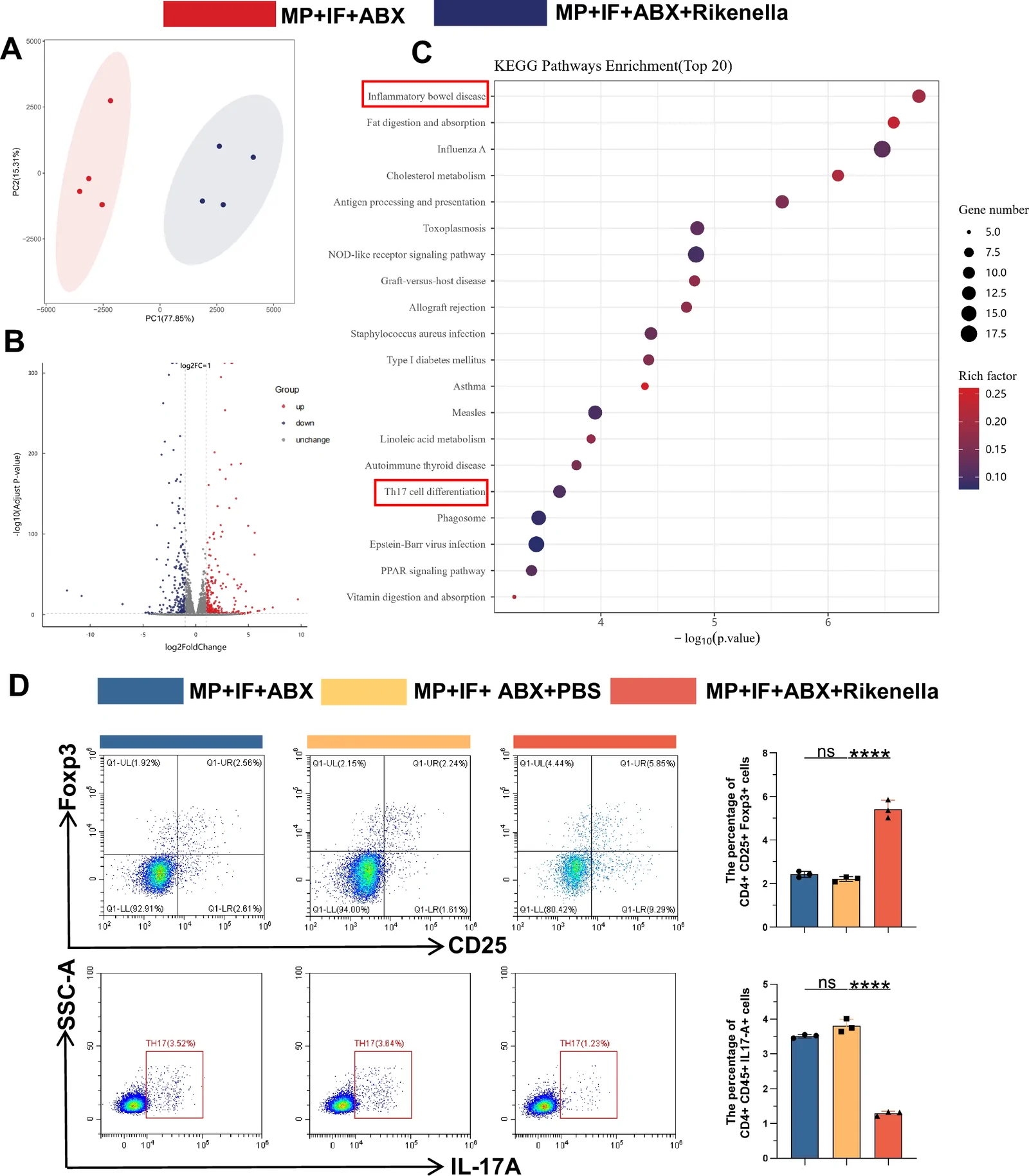
Transcriptomic analysis suggests that *Rikenella* may alleviate inflammation by regulating the Th17/Treg ratio. After establishment of the MP + IF model and subsequent antibiotic treatment, mice received *Rikenella* or PBS according to the experimental design. Colon tissues were collected for RNA sequencing, and spleens were harvested for flow cytometric analysis. **A** Principal component analysis (PCA) of transcriptomic profiles from each group (*n* = 4 mice per group). **B** Volcano map of differential genes. **C** KEGG enrichment analysis of differentially expressed genes. **D** Flow cytometry was used to detect the levels of Th17 and Treg in the spleen, *Rikenella* treatment shifted the Th17/Treg balance, showing decreased Th17 and increased Treg cells (*n* = 3 mice per group), three biologically independent mice were analyzed in each group. Three biologically independent mice were analyzed in each group. Data are presented as mean ± s.e.m. Statistical significance was determined using one-way ANOVA with multiple comparisons: *p < 0.05, **p < 0.01, ***p < 0.001, ****p < 0.0001.

Prior results demonstrated that IF intensifies MP-induced intestinal inflammation, whereas reintroduction of *Rikenella* ameliorates disease progression. To elucidate the molecular mechanisms underlying this protective effect, we conducted transcriptomic analysis of colon tissues obtained from antibiotic-treated mice subsequently administered either *Rikenella* (ABX+*Rikenella*) or vehicle control (ABX + PBS). The PCA plot revealed clear separation between the two groups, indicating substantial differences in gene expression profiles and high intra-group reproducibility (Fig. 6A). Pearson correlation coefficients exceeding 0.9 further confirmed the consistency and quality of the sequencing data (Fig. S4A). Differential expression analysis identified 998 significantly altered genes, with 373 upregulated and 625 downregulated in the *Rikenella*-treated group compared to controls. Hierarchical clustering based on these differentially expressed genes (DEGs) confirmed the distinct transcriptional landscapes between groups (Fig. S4B). The Gene Ontology (GO) enrichment analysis revealed significant alterations across biological process, molecular function, and cellular component categories, with immune-related pathways prominently enriched in the biological process domain (Fig. S4C). Kyoto Encyclopedia of Genes and Genomes (KEGG) pathway analysis identified several immune and inflammation-related pathways among the top 20 enriched categories. Notably, pathways involved in inflammatory bowel disease (IBD) and Th17 cell differentiation were significantly modulated in response to *Rikenella* treatment (Fig. 6D). Key pro-inflammatory genes, including *Il17a* and *Il21*, were downregulated following *Rikenella* supplementation, suggesting suppression of the Th17-associated inflammatory response.

To functionally validate these findings, we performed flow cytometric analysis of spleen-derived T cells, quantifying CD4^+^IL17A^+^ (Th17) and CD4^+^CD25^+^Foxp3^+^ (Treg) subsets. In the *Rikenella*-treated group, Th17 cell levels were significantly reduced, while Treg cells were markedly increased, resulting in a significant decrease in the Th17/Treg ratio (Fig. 6F Fig. S3B). These results support the hypothesis that *Rikenella* mitigates intestinal inflammation by restoring immune homeostasis, specifically through the suppression of Th17 differentiation and the promotion of Treg-mediated regulation. Collectively, these findings indicate that the depletion of *Rikenella* induced by combined MP and IF exposure disrupts the Th17/Treg balance, thereby contributing to heightened inflammation. Reintroduction of *Rikenella* restores this balance and offers a protective effect, highlighting a potential microbial target for therapeutic modulation in microplastic-related intestinal disorders.

### IF is associated with disrupted purine metabolism and altered microbe–metabolite correlations

**Fig. 7:**
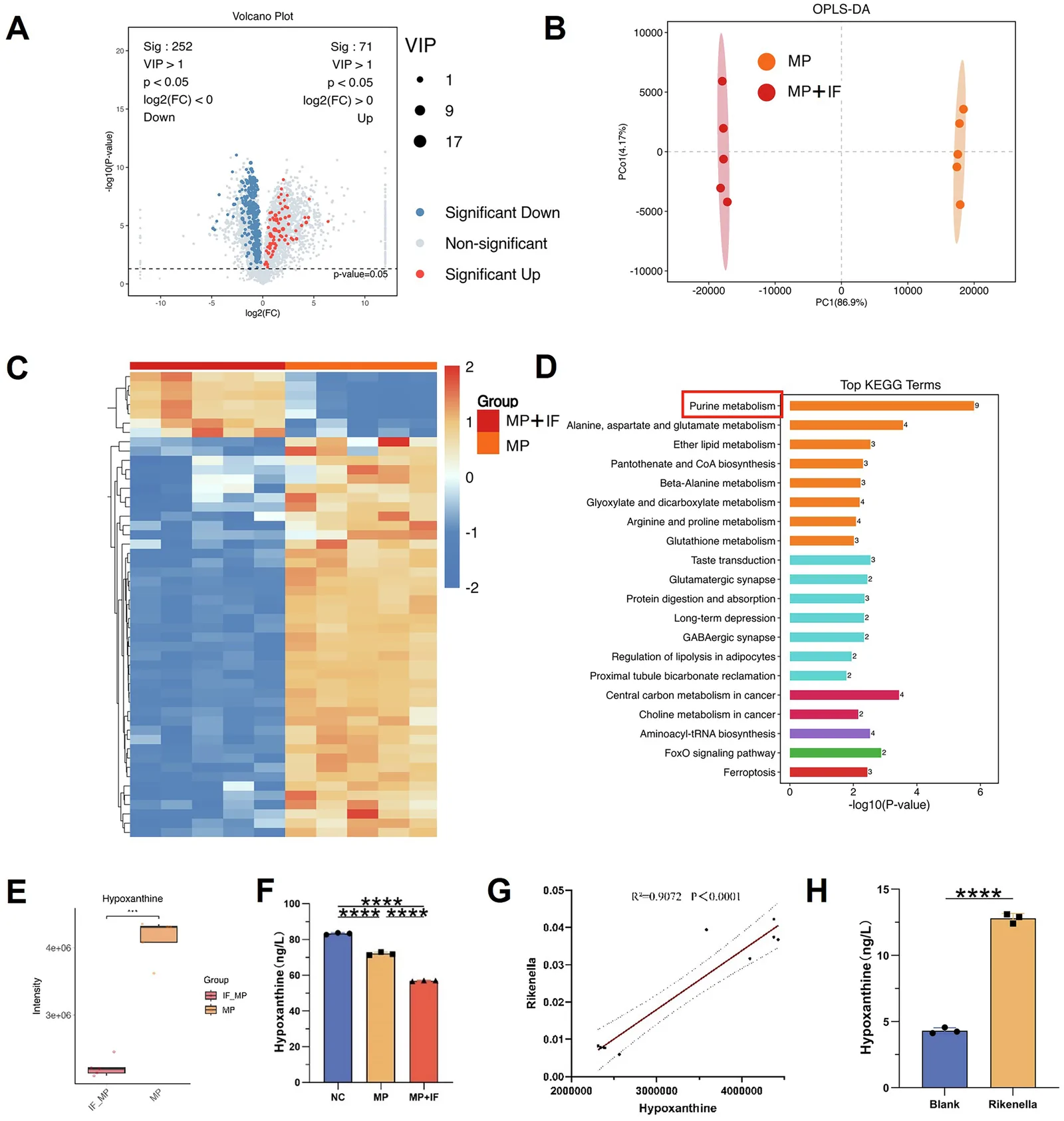
*Rikenella* regulates hypoxanthine metabolism and is involved in the progression of inflammation caused by restrictive diet. After 1 week of acclimation, mice were randomly assigned to the MP and MP + IF groups. Mice in both groups were administered 1 μm MPs by oral gavage at a dose of 10 mg/kg body weight/day for 6 consecutive weeks. Mice in the MP + IF group were additionally subjected to time-restricted feeding with an 8 h feeding window per day, with free access to water. Intestinal contents were collected for untargeted metabolomics analysis by UPLC-MS/MS. **A** Volcanic plot of differential metabolites. Volcanic plot showing differentially expressed metabolites in intestinal contents between the MP group and the MP + IF group (*n* = 5 mice per group). Red dots indicate significantly upregulated metabolites, blue dots indicate significantly downregulated metabolites, and gray dots indicate metabolites without significant difference. **B** OPLS-DA score plot showing clear metabolic separation between the MP group and the MP + IF group, indicating distinct intestinal metabolomic profiles. **C** Hierarchical clustering heatmap of differential metabolites, illustrating group-specific metabolic signatures between the MP and MP + IF groups. **D** KEGG pathway enrichment analysis of differentially expressed metabolites. Purine metabolism was among the most significantly enriched pathways. **E** Box plot of differential metabolite hypoxanthine content between the MP group and MP + IF group. **F** ELISA detection of hypoxanthine content in mouse intestinal tissue (*n* = 3 mice per group). **G** Correlation analysis showed a significant positive correlation between hypoxanthine levels and *Rikenella* abundance (*R*² = 0.9072, *P* < 0.0001). The red line represents the linear regression fit, and the black dashed line represents the 95% confidence interval. **H** Compared with the blank culture group, the content of hypoxanthine in the *Rikenella* culture supernatant was significantly increased. Data are presented as mean ± s.e.m. Statistical significance was determined using one-way ANOVA with multiple comparisons: *p < 0.05, **p < 0.01, ***p < 0.001, ****p < 0.0001.

The gut microbiota and host metabolism are intricately intertwined, with microbial communities playing a crucial role in shaping metabolite profiles that influence immune responses and inflammatory processes^28,29^. Building on previous findings that *Rikenella* mitigates MP-induced intestinal inflammation, we hypothesized that its effects may be mediated through alterations in metabolic pathways, particularly under conditions of IF. To explore this, we performed untargeted metabolomics profiling using UPLC-MS/MS on intestinal contents from mice in the MP and MP + IF exposure groups. In total, 323 differentially expressed metabolites were detected, among which 71 were significantly upregulated and 252 were downregulated in the MP + IF group compared to the MP group (Fig. 7A). OPLS-DA revealed a distinct metabolic separation between the two groups, indicating a significant shift in the intestinal metabolome due to combined MP and IF exposure (Fig. 7B). Hierarchical clustering of these metabolites, particularly the top 50 based on VIP scores, highlighted robust group-specific metabolic signatures (Fig. 7C). KEGG pathway enrichment analysis of the altered metabolites revealed significant perturbations in several metabolic pathways, with purine metabolism, the FoxO signaling pathway, and β-alanine metabolism among the most enriched. Notably, purine metabolism emerged as the most significantly disrupted pathway (Fig. 7D). Closer examination of purine pathway metabolites revealed a marked decrease in xanthine and hypoxanthine levels in the MP + IF group compared with the MP group (*P* < 0.001) (Fig. 7E). To validate these findings, we quantified hypoxanthine levels in colonic tissue homogenates via ELISA (*P* < 0.0001). The results were consistent with metabolomics data, showing significantly lower hypoxanthine concentrations in the MP + IF group (Fig. 7F).

To explore the potential relationship between *Rikenella* and hypoxanthine, we performed an integrated analysis combining 16S rRNA sequencing data and untargeted metabolomics data. Correlation analysis showed a significant positive correlation between the abundance of Hypoxanthine and *Rikenella* (Fig. 7G). Further analysis revealed that the level of Hypoxanthine in *Rikenella* culture supernatant was significantly higher than in blank medium, suggesting that *Rikenella* may be one of the potential sources of hypoxanthine(Fig. 7H). In summary, *Rikenella* may be an important bacterial genus involved in regulating hypoxanthine metabolism under combined MP and IF stress conditions, and may participate in intestinal inflammatory processes by influencing purine metabolism.

### Hypoxanthine restores immune homeostasis and ameliorates colonic inflammation

Untargeted metabolomic profiling revealed a marked reduction in hypoxanthine levels in mice subjected to both MP exposure and IF, a finding corroborated by targeted ELISA quantification. Hypoxanthine, a critical metabolite in the purine metabolism pathway, is known to play essential roles in maintaining intestinal barrier integrity and modulating immune responses. Restoration of hypoxanthine levels has been shown to attenuate inflammatory responses in various models of IBD^30^. To elucidate the immunoregulatory role of hypoxanthine, particularly in the context of MP and IF co-exposure, we established a murine model with antibiotic-induced microbiota depletion to control for microbial confounders. Following microbiota clearance, mice were assigned to one of four intervention groups: (1) MP and IF exposure with no microbial or metabolic supplementation (reference control); (2) MP and IF exposure with hypoxanthine supplementation; (3) MP and IF exposure with *Rikenella* supplementation; and (4) MP and IF exposure with combined hypoxanthine and *Rikenella* supplementation.

**Fig. 8:**
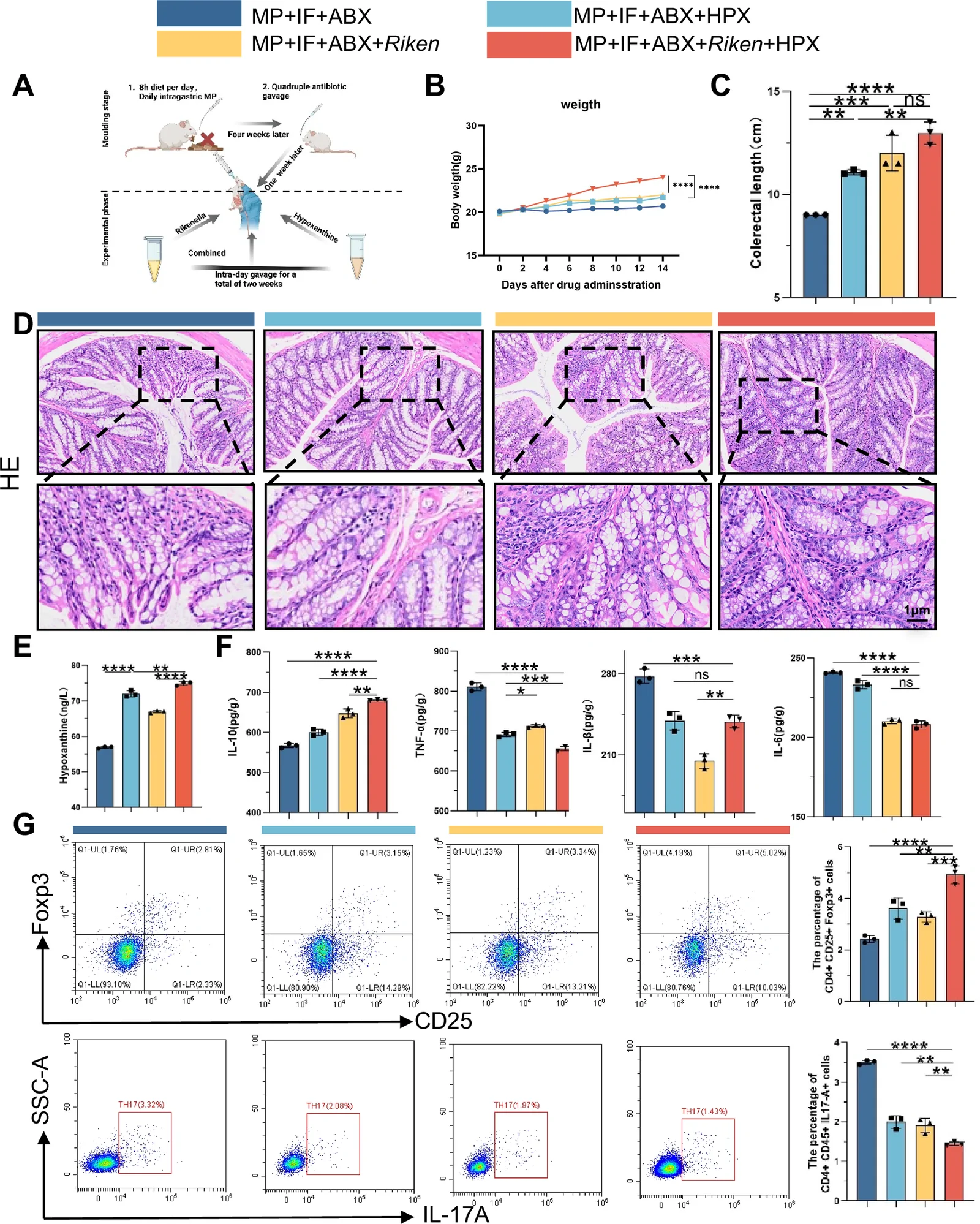
Hypoxanthine alleviates intestinal inflammation by regulating the Th17/Treg ratio. After establishment of the MP + IF mouse model, mice were treated with a broad-spectrum antibiotic cocktail by gavage to deplete the gut microbiota. The antibiotic mixture contained ampicillin, neomycin, metronidazole, and vancomycin at final concentrations of 10, 10, 10, and 5 mg/mL, respectively, and was administered at 10 μL/g body weight once daily for 7 consecutive days. After microbiota depletion, mice were assigned to the following groups according to the experimental design: MP + IF + ABX, MP + IF + ABX+hypoxanthine, MP + IF + ABX+*Rikenella*, and MP + IF + ABX+hypoxanthine+*Rikenella*. Hypoxanthine was administered by gavage at 300 mg/kg (30 mg/mL, 10 μL/g body weight) 3 times per week for 2 consecutive weeks. *Rikenella* was administered by gavage at 100 μL/mouse (1.5 × 10^9^ CFU/mL) 3 times per week for 2 consecutive weeks (*n* = 3 mice per group). **A** Model diagram of mouse modeling processing method. **B** Body weight changes of mice during the 2-week intervention period in each treatment group (*n* = 3 per group). **C** Representative images and quantitative analysis of colon length in mice from each group. Combined HPX and Rikenella treatment showed the greatest improvement in colon shortening. **D** H&E staining showed that inflammatory cell infiltration was reduced in the colonic mucosa of mice treated with Hypoxanthine and *Rikenella*. **E** ELISA detection of Hypoxanthine content in colon tissue of mice in each group. **F** ELISA quantification of colonic cytokines showed that the levels of pro-inflammatory markers (TNF-α, IL-6) were reduced and the level of anti-inflammatory cytokine (IL-10) was increased in the treatment group, IL-β showed no significant change. **G** Flow cytometry detection of Th17 and Treg ratio in the spleen, the balance of Th17 and Treg was significantly improved after treatment. Three biologically independent mice were analyzed in each group. Data are presented as mean ± s.e.m. Statistical significance was determined using one-way ANOVA with multiple comparisons: *p < 0.05, **p < 0.01, ***p < 0.001, ****p < 0.0001.

Over a 2-week intervention period, mice receiving combined hypoxanthine and *Rikenella* exhibited the most significant increases in body weight, followed by mice treated with either agent alone, relative to the reference control (*P* < 0.0001) (Fig. 8B). Comparative analysis of colon length demonstrated similar trends, with combined supplementation yielding the longest colon lengths, indicative of reduced inflammation-associated tissue shortening (Fig. 8C). H&E staining revealed substantial inflammatory infiltration in the reference group, whereas colonic tissues from hypoxanthine- and *Rikenella*-treated mice exhibited significantly attenuated inflammation, particularly in the combined treatment cohort (Fig. 8D). In addition, measurement of colonic hypoxanthine levels showed that hypoxanthine supplementation markedly increased local hypoxanthine(HPX) concentrations, as expected. Notably, *Rikenella* administration also significantly elevated colonic hypoxanthine levels compared with the MP + IF + ABX group, indicating an association between *Rikenella* supplementation and local purine metabolic changes (Fig. 8E). ELISA quantification of pro- and anti-inflammatory cytokines further supported these findings. Mice receiving hypoxanthine displayed significantly lower levels of TNF-α and IL-6, alongside increased IL-10 levels, compared with the reference control (Fig. 8F).

Flow cytometric analysis of splenic CD4^+^ T cells revealed that hypoxanthine treatment resulted in a significant reduction in Th17 cell frequencies and an increase in Treg cell populations, leading to a pronounced decrease in the Th17/Treg ratio (Fig. 8G, Fig. S3C). Moreover, colonic hypoxanthine concentrations were elevated in mice receiving *Rikenella* supplementation, suggesting a mechanistic link between this bacterial genus and the regulation of purine metabolism. Together, these findings suggest that hypoxanthine exerts anti-inflammatory effects in the context of MP and IF-induced colonic inflammation by restoring the Th17/Treg balance. *Rikenella* appears to contribute to this immune metabolic axis by modulating local hypoxanthine availability, highlighting a potential microbiota-metabolite-immune interaction underlying intestinal homeostasis.

## Discussion

A growing body of evidence indicates that oral MP exposure can disrupt intestinal homeostasis through multiple converging mechanisms, including epithelial barrier injury, oxidative stress, gut microbiota dysbiosis, and activation of inflammatory signaling pathways^17,23^. Recent reviews and experimental studies further suggest that MP toxicity is not limited to inflammation alone, but is also accompanied by substantial metabolic reprogramming within the gut environment^31^. In this study, we provide compelling evidence that IF, under conditions of MP-induced intestinal inflammation, does not confer protective effects but rather intensifies inflammatory responses and immune dysregulation. Compared to mice exposed solely to MPs, those subjected to IF exhibited exacerbated colonic damage, increased pro-inflammatory cytokine production, and a marked disturbance in immune homeostasis, characterized by a decrease in anti-inflammatory Treg cells and a concomitant increase in pro-inflammatory Th17 cells. These findings highlight a critical context-dependent aspect of IF: while traditionally recognized for its anti-inflammatory and metabolic benefits, IF may under specific environmental stressors, such as MP exposure, act as a pro-inflammatory trigger, worsening intestinal immune dysfunction

At present, the directional relationship between MP exposure and IF in driving intestinal injury remains unclear. Based on the available evidence, it is difficult to conclude whether MP is the dominant initiating factor and IF merely amplifies its toxicity, or whether fasting-induced physiological changes first increase host susceptibility to MP-associated injury. More likely, these two factors interact in a mutually reinforcing manner, such that each may exacerbate intestinal inflammatory responses under susceptible conditions. Notably, a previous study has reported that IF can aggravate MP-associated disease manifestations, although the underlying mechanisms were not explored in depth^32^. In this context, our findings provide a more mechanistic interpretation of this phenomenon by suggesting that the detrimental interaction between MP and IF may involve coordinated disturbances in gut microbial composition, purine metabolism, and immune homeostasis.

The bidirectional nature of IF’s effects on the gut and immune system is increasingly recognized. Numerous studies have underscored the beneficial aspects of IF, including improved gut microbial diversity, metabolic health, and systemic anti-inflammatory effects^12,33,34^. Time-restricted feeding has been associated with enrichment of beneficial bacterial phyla and genera, alongside weight loss and improved metabolic parameters^35^. In autoimmune and inflammatory models, IF has been shown to elevate regulatory T cell populations and suppress effector T cell responses, reducing tissue inflammation^36,37^. However, emerging evidence suggests that the immunomodulatory effects of IF are not universally beneficial and may be modulated by environmental and physiological contexts. A clinical cohort study of ulcerative colitis patients during Ramadan fasting revealed symptom exacerbation despite stable objective inflammatory markers, suggesting that IF may aggravate disease activity in specific subgroups^38^. Our data extend these findings by demonstrating in a murine model that the co-occurrence of IF and MP exposure amplifies intestinal inflammation. This is likely due to a cascade of adverse events initiated by fasting-induced gut microbiota dysbiosis, impaired epithelial barrier function, and excessive immune activation, collectively disrupting the delicate Th17/Treg balance^39^. Thus, while IF remains a promising intervention in many settings, its application should be judiciously tailored based on environmental and host-specific factors^39–41^. Compared with many previous studies in which IF showed protective effects, our model differed in that fasting was applied in the setting of chronic MP exposure, where the host intestine was already under persistent environmental stress^42^. This difference in experimental context may help explain why IF was detrimental rather than protective in the present study.

Central to our mechanistic insights is the role of gut microbiota, particularly the commensal genus *Rikenella*. 16S rRNA sequencing revealed that IF, in the context of MP exposure, significantly depletes *Rikenella* abundance. *Rikenella* has emerged as a key player in maintaining intestinal homeostasis through its support of epithelial barrier integrity and anti-inflammatory functions^43^. Reduced levels of *Rikenella* have been documented in patients with IBD and other chronic inflammatory conditions, whereas its enrichment correlates with mucosal health and disease remission^44–46^. Our functional experiments demonstrated that replenishment of *Rikenella* restored colon length, reduced histological inflammation, suppressed pro-inflammatory cytokine production, and rebalanced the Th17/Treg axis. These findings underscore the importance of microbial symbiosis in mitigating inflammation and suggest that the loss of beneficial taxa like *Rikenella* may be a critical mediator of IF-induced immune dysregulation under MP exposure.

Further exploration of underlying mechanisms implicated the purine metabolic pathway as a downstream target of *Rikenella*’s regulatory functions. Metabolomic profiling revealed a significant reduction in hypoxanthine levels in MP + IF-treated mice, a key purine metabolite with known immunomodulatory properties. Purine metabolism is integral to both energy homeostasis and immune function, with metabolites such as inosine and hypoxanthine exerting protective effects against inflammation^47,48^. Disruption of this pathway, particularly reductions in hypoxanthine, has been associated with epithelial barrier dysfunction and exacerbation of colitis^49^. Our correlation analyses and validation experiments confirmed that *Rikenella* abundance positively correlates with hypoxanthine levels, and that hypoxanthine supplementation mimics the anti-inflammatory effects of *Rikenella*, restoring Treg levels and dampening Th17-driven inflammation. Taken together, these findings suggest that *Rikenella* alleviates inflammation by maintaining purine metabolic balance and regulating the Th17/Treg ratio. IF, under MP-induced stress, disrupts this regulatory axis by depleting *Rikenella*, thereby impairing purine metabolism and exacerbating immune dysregulation. This underscores the central role of the microbiota/metabolism/immunity axis in mediating the effects of dietary interventions.

In conclusion, our study reveals that the immunological consequences of IF are highly context-dependent. While IF offers substantial therapeutic potential, its effects may be deleterious under conditions of environmental stress, such as MP exposure. We highlight the pivotal role of *Rikenella* and purine metabolism in modulating intestinal immunity, providing mechanistic insight into how microbial and metabolic networks mediate the dual effects of IF. These findings underscore the need for precision dietary strategies that account for host–microbe–environment interactions. Future investigations should focus on delineating the conditions under which IF is beneficial versus harmful, with an emphasis on integrating microbiome-targeted therapies to optimize outcomes in inflammatory diseases. Our work contributes to a deeper understanding of the complex interplay among diet, microbiota, metabolism, and immunity, advancing the field of personalized nutrition and gut health.

## Materials and methods

### Materials

Two types of PS microplastics (ZhiChuan, Jiangsu, China) were used: standard MPs (1 μm) for toxicity assays and red fluorescent MPs (1 μm, Ex/Em: 520/580 nm) for cellular uptake visualization. For 1 μm PS microplastics, 1 mg corresponds to approximately 1.53 × 10⁹ particles. MPs were characterized by SEM (Sigma 300, ZEISS), particle size analyzer (Mastersizer 2000, Malvern), and FTIR (IRTracer 100, Shimadzu). Flow cytometry reagents (mouse FcR blocker) from Elabscience (Wuhan).

### Animal husbandry

Specific Pathogen-Free (SPF) BALB/cmice (4 weeks old, male) were obtained from the Anhui Experimental Animal Center (Hefei, Anhui, China). All mice were housed under controlled conditions at 22–26 °C, with appropriate humidity and a 12-h light/dark cycle, and were acclimated for 1 week prior to experiments. This setup provided a controlled environment to study the effects of microplastic exposure and intermittent fasting on gut health and inflammation in the murine model. All experimental procedures were performed in accordance with the guidelines of the Animal Care and Use Committee of Anhui Medical University (Approval No. 20242418 and LLSC).

### Cell culture

Human colon epithelial cells (NCM460; Warner Bio, Wuhan, China) were cultured in RPMI 1640 with 10% FBS and antibiotics (Gibco), maintained at 37 °C with 5% CO₂. Upon reaching >90% confluence, cells were seeded into 6-well plates for experiments. Cells used in the experiments were maintained under standard culture conditions and used between passages 4 and 6.

### Construct mouse models

After 1-week acclimation, mice were randomly divided into four groups (*n* = 3 mice per group): NC, MP, IF, and MP + IF. Body weights were measured every 2 days. The MP and MP + IF groups received daily gavage of PS microplastics, the oral dose was calculated based on the daily body weight of the mice and was 10 mg/kg (10 mg/kg ≈ 1.53 × 10¹⁰ particles/kg); controls received PBS. IF groups had 8-h feeding daily with fasting otherwise (water unrestricted). After 6 weeks, mice were weighed, fasted 8 h, euthanized (pentobarbital anesthesia and cervical dislocation), and samples (serum, feces, organs) were collected for analysis. For the rescue experiments, the MP + IF mouse model was established first, followed by antibiotic treatment for gut microbiota depletion. To establish the gut microbiota depletion model, mice in the ABX group were intragastrically administered a cocktail of antibiotics containing ampicillin, neomycin, metronidazole, and vancomycin at final concentrations of 10, 10, 10, and 5 mg/mL, respectively. The antibiotic mixture was gavaged once daily at 10 μL/g body weight for 7 consecutive days, corresponding to doses of 100, 100, 100, and 50 mg/kg, respectively. Control mice received an equal volume of sterile water. After ABX treatment, mice were subjected to *Rikenella* or hypoxanthine intervention according to the experimental design. For *Rikenella* treatment, *Rikenella* was cultured anaerobically, collected, and resuspended in sterile PBS to a final concentration of 1.5 × 10^9^ CFU/mL. Each mouse received 100 μL of the bacterial suspension by gavage, three times per week for 2 weeks, corresponding to 1.5 × 10^8^ CFU per mouse per administration. For hypoxanthine treatment, hypoxanthine was prepared in 0.5% CMC-Na at 30 mg/mL and administered by gavage at 10 μL/g body weight (300 mg/kg per administration), three times per week for 2 weeks. Control mice received an equal volume of vehicle.

### Cellular uptake of fluorescently labeled PS microplastics

NCM460 cells were seeded in 6-well plates and cultured for 24 h. At approximately 80% confluence, the cells were co-incubated with 1 μm red fluorescent PS microplastics at a concentration of 1 mg/mL for another 24 h. Based on the density of polystyrene (1.05 g/cm³) and assuming spherical particles, 1 mg of 1 μm PS microplastics corresponds to approximately 1.53 × 10⁹ particles, resulting in a particle concentration of 1.53 × 10⁹ particles/mL in the exposure medium.

The total exposure volume per well was 5 mL, and the number of cells was approximately 1.5 × 10⁶ cells, corresponding to approximately 5100 particles per cell. After incubation, cells were washed with PBS to remove unengulfed particles. Red fluorescence and DAPI images were then captured using an Olympus inverted fluorescence microscope to evaluate microplastic uptake by the cells.

### Histopathological staining

To assess inflammation in the colon, histopathological examination was conducted on mouse colon samples. The tissues were first fixed in 4% paraformaldehyde to preserve their structure. After fixation, the samples underwent dehydration, paraffin embedding, and were sectioned into 4 μm thick slices. Hematoxylin and eosin (HE) staining was performed using reagents provided by Solarbio (Beijing, China) to visualize cellular and tissue morphology. Finally, the stained tissue sections were observed and imaged using a Leica upright microscope (DM6B, Leica, Germany).

### Inflammatory factors are detected by ELISA

Cytokine levels were measured in colon tissue homogenates using ELISA kits according to the manufacturer’s instructions. Colon tissue cytokine levels (TNF-α, IL-1β, IL-6, IL-10) were measured by ELISA (Ruixinbio, China) following the manufacturer’s instructions. OD values were read at 450 nm (BioTek), and concentrations were calculated using standard curves to assess inflammation.

### Detection of biomarkers of oxidative stress

To assess the levels of oxidative stress biomarkers in mouse colon tissues, homogenates were prepared following the manufacturer’s instructions. The levels of reduced glutathione (GSH) and oxidized glutathione (GSSG) were measured using colorimetric assays (GSH and GSSG Assay Kit, S0053, Beyotime, Shanghai, China). Additionally, malondialdehyde (MDA) levels were determined using a microplate reader (MDA Assay Kit, BC0025, Solarbio). These measurements provided insights into the oxidative stress status of the colon tissues, enabling a comprehensive evaluation of the oxidative damage and antioxidant capacity.

### Single-cell sequencing data analysis

The GSE226553 dataset from the GEO database was subjected to single-cell analysis, which includes scRNA-seq data from BALB/c mice (age 5 weeks) and ICR mice (age 6 months) treated with microplastics for 1 week^50^.

### Data preprocessing

Data processing largely followed the pipeline described in the original study and standard workflows widely adopted in recent single-cell studies. The raw gene–barcode matrix was analyzed using the Seurat package in R. Strict quality control was applied to remove low-quality cells. Cells with fewer than 200 or more than 7000 detected genes were excluded to remove empty droplets and potential doublets. Cells with mitochondrial gene content greater than 10% were filtered out to eliminate low-quality or apoptotic cells. In addition, the proportions of ribosomal genes (>35%) and hemoglobin genes (>3%) were evaluated to minimize technical bias and potential contamination from erythrocytes. Potential doublets were identified and removed using Doublet Finder, with the expected doublet rate estimated according to cell loading density and sequencing platform recommendations. Only genes expressed in at least three cells were retained for downstream analysis. Normalization and variance stabilization were performed using the SCTransform method implemented in Seurat, which is based on a regularized negative binomial regression model. The percentage of mitochondrial genes was regressed out during normalization to reduce technical variation in the expression matrix. The top 2000 highly variable genes were selected for PCA, and the first 30 principal components were retained for downstream analyses based on the elbow plot and the cumulative variance explained.

To identify the top marker genes for each cluster, differential expression analysis was conducted using the FindAllMarkers function, focusing on positive marker genes—those specifically highly expressed in a particular cell population. The filtering criteria were set as follows: genes must be expressed in at least 25% of cells within the cluster, and the log_2_ fold change must be greater than 0.25. The results were grouped by cluster and sorted in descending order based on the average log2 fold change. The top 30 marker genes for each cluster were then extracted. These top 30 genes were imported into the CellMarker 2.0 database for cell type annotation^51^. The annotation results were compared with those generated by the SingleR package, and the most appropriate annotation was selected^52^. This annotation was subsequently added to the Seurat object for downstream analysis.

### Cell trajectory analysis

We performed pseudotime analysis on the single-cell transcriptome data using the Monocle package^53^. First, the estimate Size Factors and estimate Dispersions functions were used for data normalization and dispersion estimation. Next, the detect Genes function was applied to identify genes expressed at a level of at least 0.1, and genes expressed in at least 10 cells were selected as the basis for analysis. To select ordering genes, highly variable genes were extracted and designated as ordering genes. Subsequently, differential expression analysis was conducted on the expressed genes using a full model formula based on cell type, and genes with a *q*-value (adjusted *p*-value) less than 0.01 were selected as significantly DEGs, sorted in ascending order by *q*-value. After selecting these significant differential genes as ordering genes, the DDRTree algorithm was used to reduce the data to two dimensions, and a cell pseudotime trajectory was constructed to infer the developmental dynamics of the cells.

### Cell-to-cell communication analysis

In this study, the CellChat package^54^ was utilized to construct and compare intercellular communication networks across different groups. Initially, the cells in the dataset were categorized into the MP group and the NC group, and a distinct CellChat object was generated for each group based on cell type. Leveraging the built-in mouse database within CellChat, each group underwent analysis through the following steps: data subsetting, identification of highly expressed genes and interaction pairs, computation of intercellular communication probabilities and their pathway-level probabilities, summarization of network data, and calculation of network centrality. The CellChat objects for both the MP and NC groups were subsequently saved for further analysis. To examine the differences in intercellular communication between the MP and NC groups, the CellChat objects for both groups were merged, and multiple comparative analyses were performed, including the comparison of total interactions and weights, as well as communication heatmap analysis.

### Single-cell enrichment analysis

We selected the highly expressed genes from cell subpopulations and performed GSEA enrichment analysis using the BP dataset from the GO database as a reference.

### Th17/Treg cell ratio determination

Th17/Treg cells were quantified by flow cytometry. Mouse spleen lymphocytes were isolated, RBC lysed, washed with PBS, and blocked with FcR blocker. Cells were stained with surface antibodies (CD45-PB450, CD4-FITC, CD8-APC, CD3-PerCP/Cyanine5.5, CD25-PE), fixed, permeabilized, and incubated with intracellular antibodies (Foxp3-APC, IL-17A-PE/Cyanine7). All antibodies used in this study were purchased from Elabscience. Analysis was performed using a Beckman Coulter flow cytometer and FlowJo software.

### Rikenella culture

The modified PYG medium was prepared according to DSMZ Medium 104 (PYG medium, modified). After dissolution in hot water and sterilization by autoclaving, hemin chloride, vitamin K1, and cysteine hydrochloride were added to the medium in an anaerobic glove box. The medium was then aliquoted into test tubes and kept in the glove box for 24 h to ensure oxygen removal. *Rikenella* microfusus DSM 15922 (*Rikenella*) was inoculated into the medium and cultured anaerobically at 37 °C for 24 h. After determining the bacterial concentration using the McFarland turbidity method, the suspension was adjusted to the desired concentration^55,56^.

### Tissue immunofluorescence

Colon tissues collected from mice were fixed in 4% paraformaldehyde, embedded in paraffin, and cut into 3 μm-thick sections. The sections were first deparaffinized with xylene and rehydrated through a graded ethanol series to distilled water. Endogenous peroxidase activity was then blocked using a blocking reagent (AR1108, Boster, China). For antigen retrieval, the sections were heated in antigen retrieval buffer using a microwave oven and then allowed to cool naturally to room temperature. After rinsing with phosphate-buffered saline (PBS), the tissue sections were incubated with 5% bovine serum albumin (BSA) for 30 min at room temperature to minimize nonspecific binding. The sections were subsequently incubated overnight at 4 °C with primary antibodies against CD4, CD25, and IL-17A (1:200 dilution) and FOXP3 (1:100 dilution). After incubation, the sections were washed three times with PBS to remove excess primary antibodies.

Next, the sections were incubated with a FITC-conjugated secondary antibody (Biosharp, China) for 2 h at room temperature in the dark. Following additional PBS washes, nuclei were counterstained with a DAPI-containing antifade mounting medium (P0131, Beyotime Biotechnology, China). Finally, the stained sections were observed and photographed using a Leica fluorescence microscope.

### Untargeted metabolomics

Raw LC–MS data were processed using Progenesis QI software (version 2.3, Nonlinear Dynamics, UK). Data preprocessing included spectral deconvolution, peak detection, retention time alignment within a 0.1 min window, and signal intensity normalization across all samples. Mass spectral features were extracted using stringent mass tolerance settings, with a precursor ion tolerance of <5 ppm and a fragment ion tolerance of <10 ppm. Only fragment ions with a relative abundance greater than 5% were retained for subsequent analysis.

Metabolite identification was performed by matching the detected features against several public metabolite databases, including the KEGG database (Saccharomyces cerevisiae, budding yeast) (https://www.kegg.jp), the Human Metabolome Database (HMDB, version 4.0) (https://hmdb.ca), LIPID MAPS (https://www.lipidmaps.org), and METLIN (https://metlin.scripps.edu). In addition, a LuMet-Animal local database (version 3.0, OE Biotech, Shanghai, China; https://www.oebiotech.com) was used to assist metabolite annotation. This curated database integrates metabolite structural information and MS/MS spectral data to improve the confidence of metabolite identification^57^.

To assess the overall variation among samples, PCA was conducted. Furthermore, partial least squares discriminant analysis (PLS-DA) and orthogonal partial least squares discriminant analysis (OPLS-DA) were performed to identify differential metabolites between groups. Model robustness was evaluated using 7-fold cross-validation and 200 permutation tests. Metabolites with a variable importance in projection (VIP) value >1.0 and *p* < 0.05 were considered significantly different^58^.

### 16S rRNA sequencing of stool samples

Genomic DNA was extracted from samples using the Mag Pure Soil DNA LQ Kit according to the manufacturer’s instructions. DNA concentration and purity were evaluated using a NanoDrop 2000 spectrophotometer, and DNA integrity was verified by agarose gel electrophoresis.

The V3–V4 region of the bacterial 16S rRNA gene was amplified using the universal primers 343F (5′-TACGGRAGGCAGCAG-3′) and 798R (5′-AGGGTATCTAATCCT-3′) with Takara Ex Taq DNA polymerase. Barcoded primers were used to allow multiplex sequencing of different samples. PCR amplification products were verified by agarose gel electrophoresis and purified using AM Pure XP magnetic beads. The purified amplicons were used as templates for a second round of PCR amplification. After purification, the libraries were quantified using the Qubit dsDNA Assay Kit. Libraries from different samples were pooled at equimolar concentrations before sequencing. Sequencing was performed on an Illumina NovaSeq 6000 platform, generating paired-end reads of 250 bp.

Raw sequencing reads were processed to remove primer sequences and low-quality reads. After quality filtering, paired-end reads were merged and chimeric sequences were removed to obtain high-quality sequences. The resulting sequences were clustered to generate ASV tables. Taxonomic classification was performed by comparing representative sequences against the SILVA database to determine microbial composition.

### Transcriptome sequencing

Total RNA was extracted using TRIzol reagent according to the manufacturer’s instructions. RNA quality was comprehensively evaluated based on concentration, total amount, volume, and RNA integrity number (RIN) according to the quality control report. RNA concentration and purity were measured using a NanoDrop ND-2000 spectrophotometer, while RNA integrity was further assessed using an Agilent Bioanalyzer system. Only qualified RNA samples were used for library construction.

RNA sequencing libraries were prepared following the standard RNA-seq library preparation protocol, with an insert size of 300–400 bp. Libraries were pooled according to the proportion of the required sequencing data volume for each library and then subjected to paired-end sequencing on an Illumina Nova Seq platform. Raw sequencing reads were processed to remove adapter sequences, low-quality reads, and reads containing excessive ambiguous bases. The resulting clean reads were aligned to the mouse reference genome (GRCm39) using HISAT2. Gene expression levels were quantified using Feature Counts based on the number of reads mapped to each gene.

Differential gene expression analysis between groups was performed in R using the DESeq2 package (version 1.34.0). Raw read count data were normalized, and dispersion was estimated using the DESeq2 pipeline. A negative binomial generalized linear model was fitted for each gene, and the built-in Wald test was used to calculate raw *P* values. The Benjamini–Hochberg method was then applied for multiple testing correction to obtain adjusted *P* values (*Padj*). Genes with an absolute log2 fold change greater than 1 and a *Padj* value less than 0.05 were considered significantly differentially expressed.

To explore the potential biological functions of the DEGs identified by RNA-seq, GO functional enrichment and KEGG pathway analyses were conducted. GO enrichment was used to annotate the DEGs in terms of biological process, molecular function, and cellular component, whereas KEGG analysis was performed to identify signaling pathways significantly associated with the treatment.

## Supplementary information

Supplementary figures

## Data Availability

The data supporting the findings of this study are openly available as follows: **Single-cell sequencing data:** The single-cell sequencing dataset GSE226553 was obtained from the GEO database. **The 16S rRNA gene sequencing data** have been deposited in the NCBI Sequence Read Archive under accession number PRJNA1091122. **The transcriptomic data** are available under accession number PRJNA1091124. **The integrated multi-omics dataset** has been deposited in the China National GeneBank DataBase under project number CNP0005275. All datasets are publicly accessible and were deposited in recognized community repositories. Additional materials including the technical appendix, statistical code, and raw data are available from the corresponding author upon reasonable request (email: 11718242@zju.edu.cn). No individual consent for data sharing was obtained, but all presented data are fully anonymized and pose minimal risk of participant identification.
