# Supplementary material for: Intermittent fasting exacerbates microplastic-induced gut inflammation via Rikenella-mediated regulation of purine metabolism and Th17/Treg balance
Source: NPJ Biofilms Microbiomes. 2026 Jun 15;12:206. doi: 10.1038/s41522-026-01016-5 (PMC13677689; doi:10.1038/s41522-026-01016-5)
Supplement: Supplementary file 1 [file 41522_2026_1016_MOESM1_ESM.pdf]

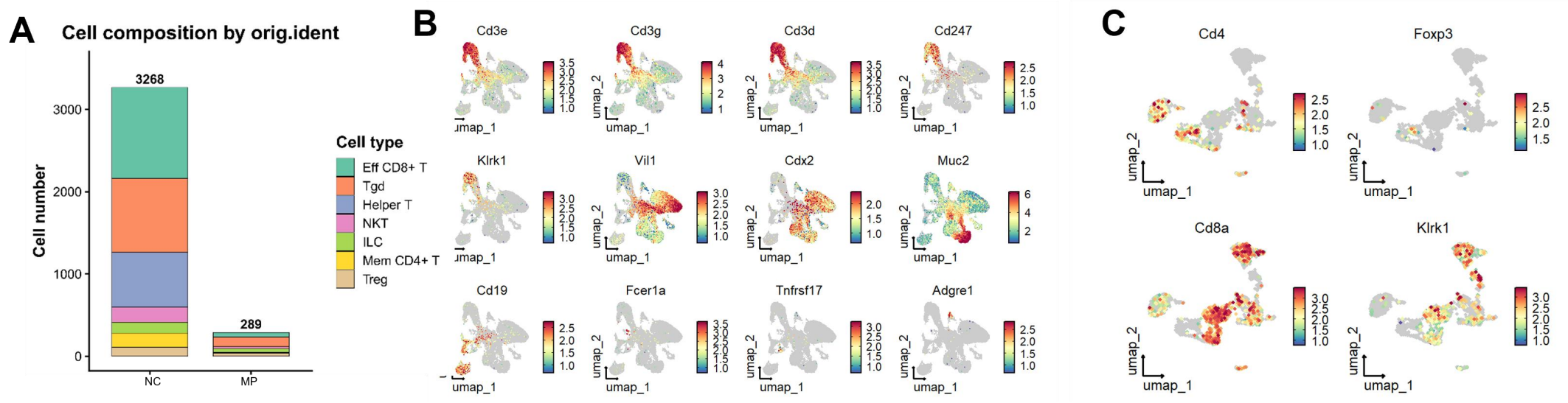

Fig .S1 |Overview of cell type composition and marker gene expression in the MP and NC groups

A, Proportions of Different Cell Types in the MP and NC Groups

B, C, Marker Gene Distribution Maps.

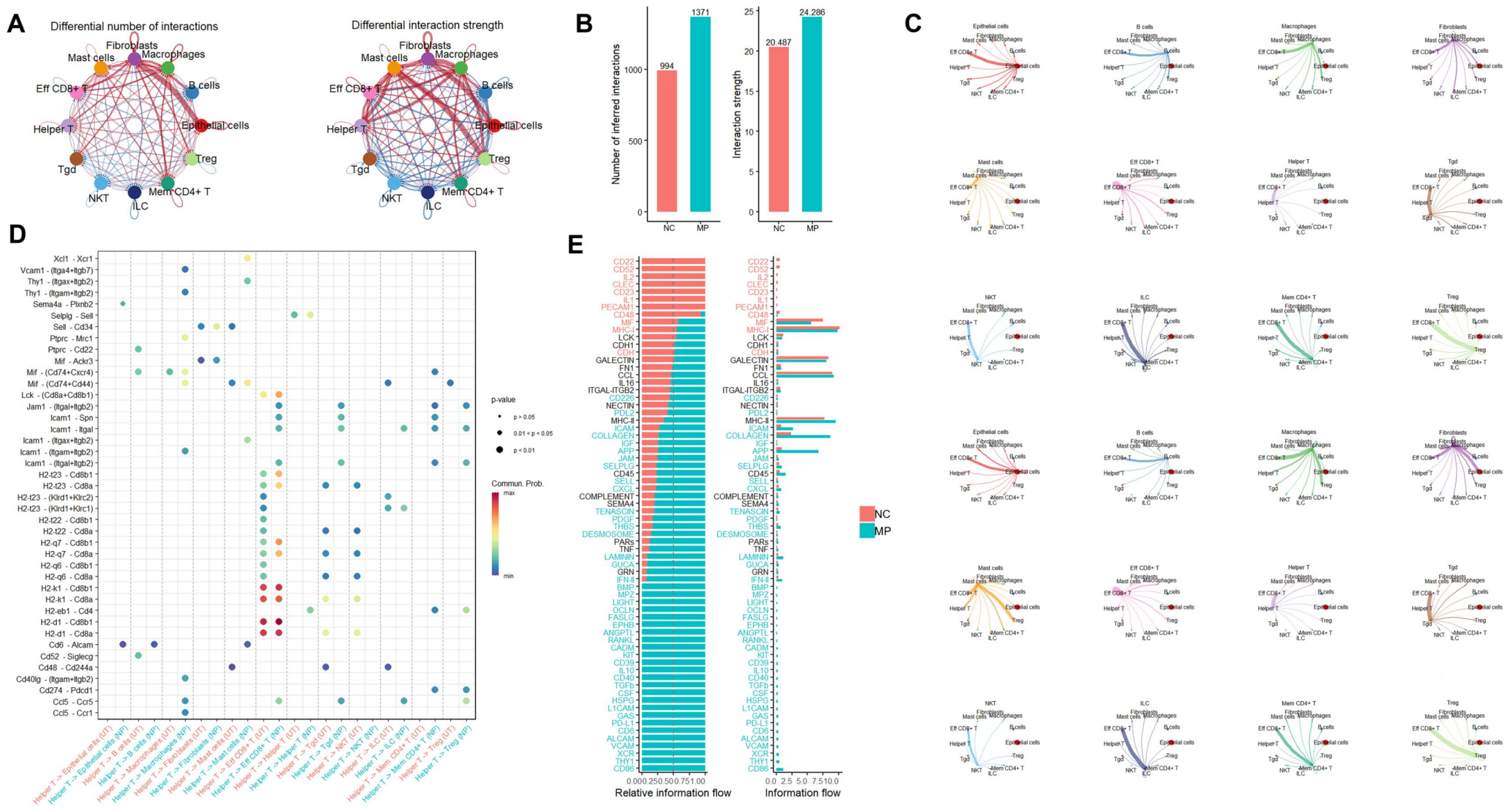

Fig . S2 |Cell-to-Cell Communication

A, Circular plot visually displaying the interaction quantity or total interaction strength (represented by weights) between any two cell populations.

B, Bar graph showing the differences in interaction quantity or total interaction strength between cell populations in the NC and MP groups.

C, Shell plot visually presenting the interaction strength between any two cell populations.

D, Comparative analysis of communication probability mediated by ligand-receptor (L-R) pairs in specific cell populations.

E, Inferring the overall information flow differences in the network between the MP and NC groups, with significant (differential) signaling pathways ranked, where the left side shows relative proportions and the right side displays absolute values.

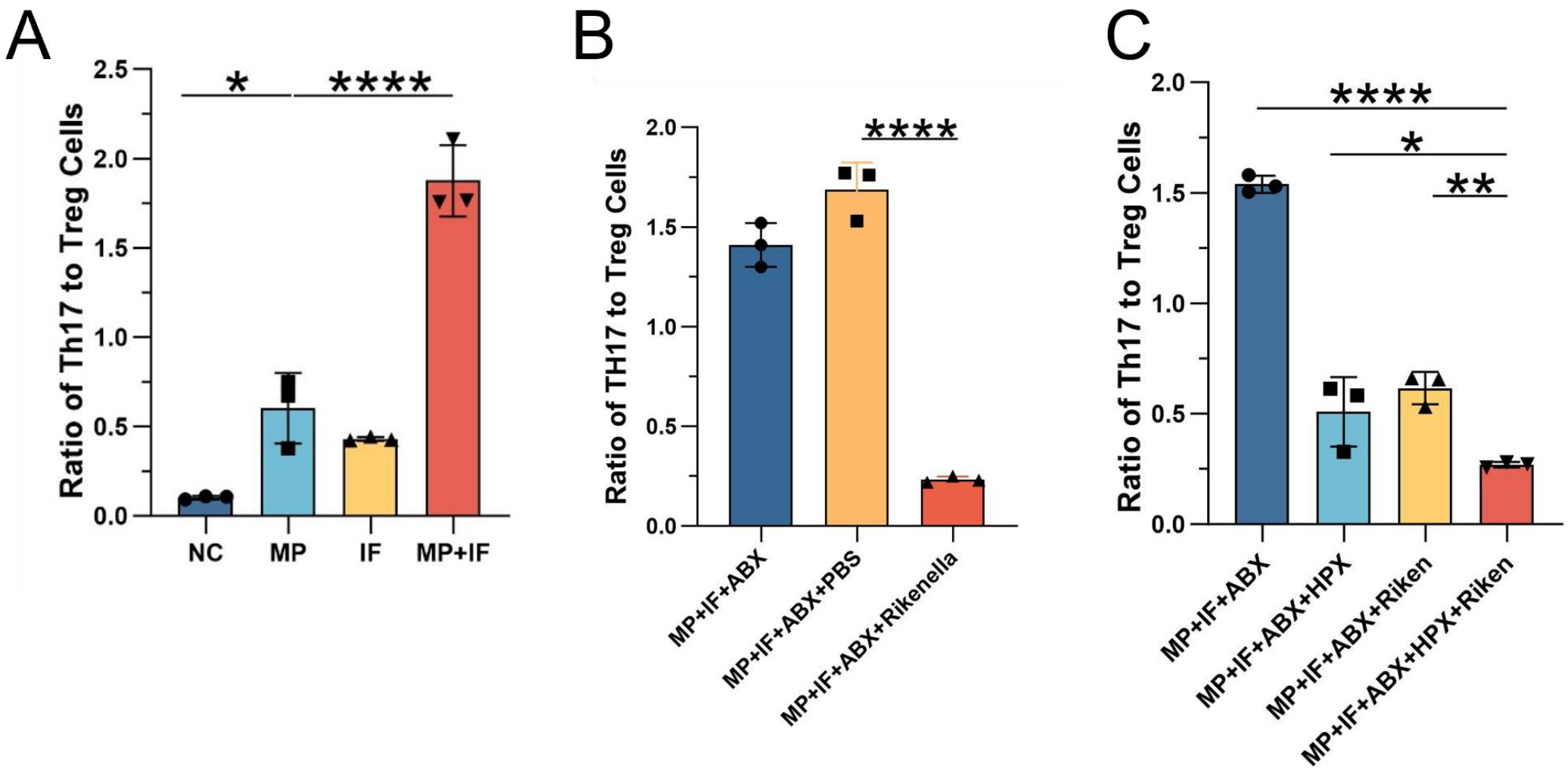

Figure. S3|Th17/Treg ratio and variance analysis across groups.

A ,The ratio of Th17 to Treg cells in NC, MP, IF, and MP+IF groups. MP+IF exposure significantly increased the Th17/Treg imbalance compared with other groups.

B, *Rikenella* administration markedly reduced the imbalance.

C, Th17/Treg ratio in MP+IF+ABX-treated groups with or without HPX and/or *Rikenella* supplementation.

Data are presented as mean ± s.e.m. Statistical significance was determined using one-way ANOVA with multiple comparisons: *$p < 0.05$, **$p < 0.01$, ***$p < 0.001$, ****$p < 0.0001$

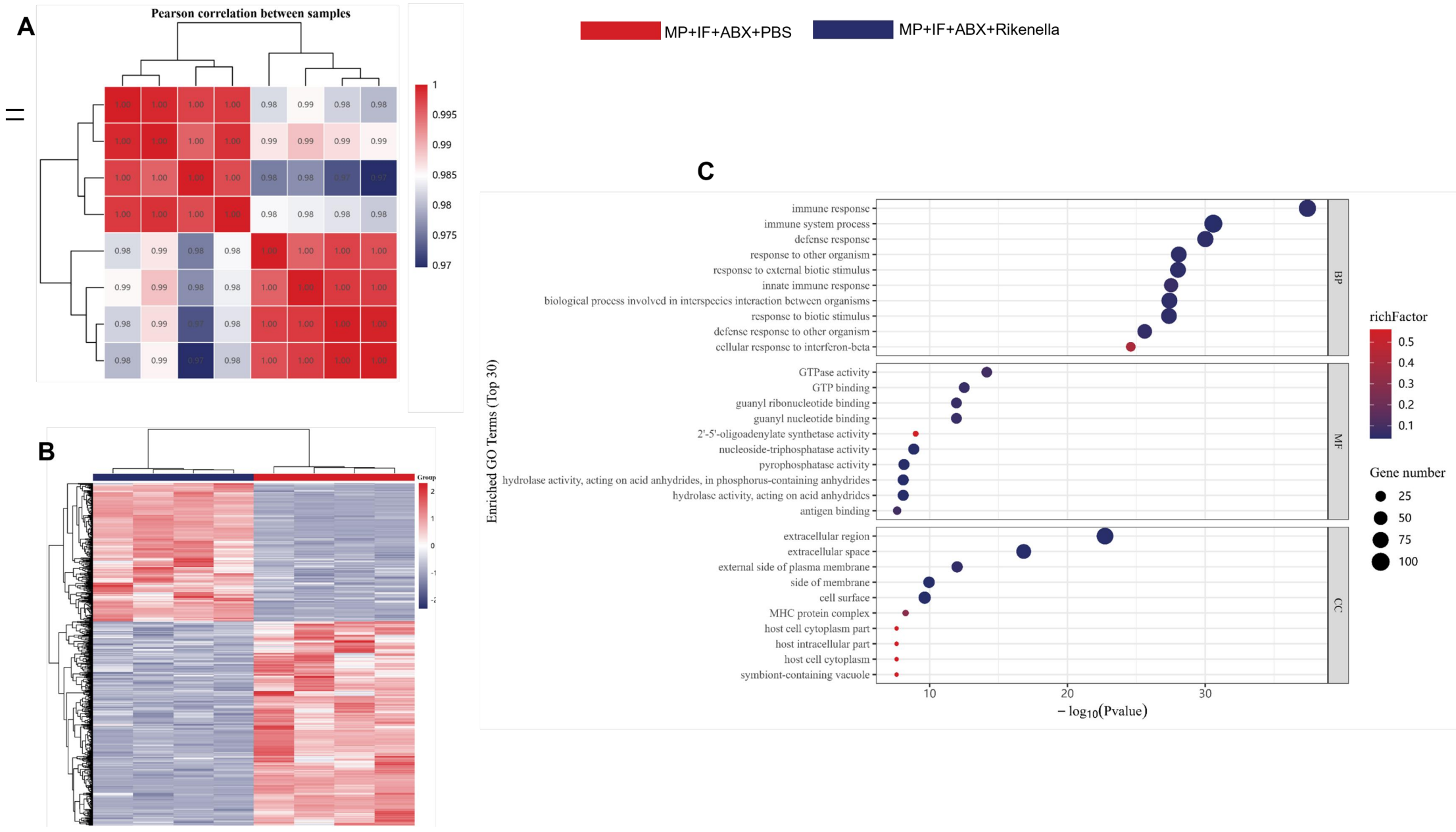

Fig .S4| Transcriptomic profiling and GO enrichment analysis of colon tissues from ABX-treated mice with or without *Rikenella* supplementation.

A, Pearson correlation heatmap showing good reproducibility among samples.

B, Hierarchical clustering heatmap of differentially expressed genes between the ABX+*Rikenella* and ABX+PBS groups.

C, GO enrichment analysis of differentially expressed genes. Dot size indicates gene number, and dot color indicates rich factor.
